# Supplementary material for: An arbitrary-spectrum spatial visual stimulator for vision research
Source: eLife. 2019 Sep 23;8:e48779. doi: 10.7554/eLife.48779 (PMC6783264; doi:10.7554/eLife.48779)
Supplement: Supplementary file 1. [file elife-48779-supp1.docx]

Supplementary File 1

| Part | Description (link) | Company | Item number |
| --- | --- | --- | --- |
| LCr | [DLP® LightCrafter™ E4500 MKII™ UV(385nm/405nm) + Blue(460nm) + Green(520nm)](https://www.ekbtechnologies.com/e-store/dlp-lightcrafter-e4500-mkii-uv-385nm-405nm-blue-460nm-green-520nm) | EKB Technologies Ltd. | DPM-E4500UVBGMKII |
| DM_M_ | Dichroic mirror mouse (custom made, *cf*. Figure 2 – figure supplement 3) | AHF Analyse-  technik AG | F73-063_z400-580-890 |
| M 00 | [UVFS Plate Beamsplitter](https://www.thorlabs.de/thorproduct.cfm?partnumber=BSX16) | Thorlabs | BSX16 |
| M 01, 02 | Silver mirrors |  |  |
| CM | [Cold Light Mirror KS 93 / 45°](https://www.qioptiq-shop.com/en/Precision-Optics/Plano-Optics/Filters/Cold-Light-Mirror-KS-93-45.html?listtype=search&searchparam=cold%20mirror&listtype=search#COLD%2520MIRROR) | Qioptiq Photonics GmbH & Co KG | G380255033 |
| Lens 05 | [ACA-254 Achromatic Doublet f = 50 mm](https://www.thorlabs.de/thorproduct.cfm?partnumber=ACA254-050-A) | Thorlabs | ACA254-050-A |
| Lens 06 | [AC-508 Achromatic Doublet](https://www.thorlabs.de/thorproduct.cfm?partnumber=AC508-100-A-ML) | Thorlabs | AC508-100-A-ML |
| BP 06 | [Band pass filter 380-407/ 562-589](https://www.ahf.de/produkte/spektralanalytik-photonik/optische-filter/einzelfilter/bandpass-filter/multiband-filter/2759/dapi/texas-red-et-dualband-anregungsfilter) | AHF Analyse-  technik AG | F59-003 |
| z stage | [13 mm Travel Vertical Translation Stage](https://www.thorlabs.com/thorproduct.cfm?partnumber=MVS005/M) | Thorlabs | MVS005/M |
| x or y stage | [13 mm Translation Stage](https://www.thorlabs.com/thorproduct.cfm?partnumber=MT1B/M#ad-image-0) | Thorlabs | MT1B/M |
